# Strengthening Early Nursing Practice: Psychometric Validation of the Turkish Version of the Professional Interpersonal Competence Scale

**DOI:** 10.1155/jonm/1343955

**Published:** 2026-05-07

**Authors:** Cemal Özalp

**Affiliations:** ^1^ Department of Health Care Services, Muş Alparslan University, Muş, Türkiye, alparslan.edu.tr

**Keywords:** beginning nurse, cultural adaptation, interpersonal competence, nurses, scale

## Abstract

**Objective:**

To culturally adapt the Professional Interpersonal Competence Evaluation Scale for novice nurses in Turkiye.

**Background:**

This methodological study aimed to translate, culturally adapt, and evaluate the psychometric properties of the Professional Interpersonal Competence Evaluation Scale for novice nurses.

**Study Design and Methods:**

This methodological study was conducted with 450 nurses working in two public hospitals between October and December 2024. The construct validity of the scale was evaluated using exploratory factor analysis (EFA) and confirmatory factor analysis (CFA). Reliability was assessed using Cronbach’s alpha, McDonald’s omega, and split‐half reliability coefficients.

**Results:**

The Kaiser–Meyer–Olkin value was 0.929, and Bartlett’s test of sphericity was significant (*χ* ^2^ = 6852.706, *p* < 0.001), indicating sampling adequacy for factor analysis. EFA revealed a two‐factor structure consisting of 26 items, explaining 44.485% of the total variance, with factor loadings ranging from 0.403 to 0.789. CFA results supported the two‐factor model with acceptable fit indices (*χ* ^2^/df = 4.62, RMSEA = 0.078, CFI = 0.94, TLI = 0.92). Item‐total correlations ranged from 0.353 to 0.753 (*p* < 0.001). The internal consistency of the scale was high (Cronbach’s *α* = 0.945; McDonald’s *ω* = 0.948).

**Conclusion:**

The results indicate that the Professional Interpersonal Competence Evaluation Scale for novice nurses is a reliable and valid evaluation tool for Turkiye.

## 1. Introduction

Nurses, who make up a large proportion of health workers, have a vital role in how health actions are organized and implemented at both the frontline and management levels [1]. Although the number of nurses is increasing today, the turnover rates of new nurses are higher compared to those of experienced nurses [2]. The decrease in the number of nurses has been reported as a situation that reduces the quality of health services [3]. Early resignation affects nurses and hospitals. Lack of human resources can mean that other nurses’ workloads increase at significant levels, resulting in lower quality care and higher risks. High resignation rates and the shortage of nurses cause serious problems in nursing and healthcare [4].

Newly graduated nurses often lack the confidence and sometimes clinical competence needed to provide safe care to patients [5]. Benner (1982) [6] addressed this problem as part of the Novice‐to‐Expert Theory and stated that new nurses might lack the needed skills and experience to start safe practice in the patient care settings [7]. Like every new university graduate, new graduate nurses experience fear, anxiety, communication problems, failure, and insecurity in the process of adaptation to work based on their changing roles and responsibilities. This might lead to an increase in clinical error rates in the clinical environment where nurses are located, cause harm to patients, and even reflect on hospital cost‐effectiveness. Newly graduated nurses are constantly made to feel the distinction between senior and beginning, experiencing communication problems, being constantly scolded and searched for deficiencies, and changing the service where they work due to lack of personnel and not being valued. They have fear of making mistakes in patient care, inability to adapt to shift work in the first years of clinical practice. It is stated that they experience stress and lack of self‐confidence in the first years of their profession due to reasons such as difficulties in transferring theoretical knowledge to practice, negative mood caused by being alone if they start working in a different city, patients and patient relatives, inability to adopt workplace culture, and inability to adapt to high technology [8].

Nursing necessitates a higher level of relationship building and communication skills. Some nurses lack the social maturity needed to communicate with colleagues, which means that such nurses probably find their work challenging; they might find it difficult to communicate with patients and staff or might face difficulties providing an appropriate level of care. These problems emphasize the relationship between the sociability of novice nurses and their early resignation [9]. In addition, conducted by Kankaya and colleagues, the difficulties experienced by novice nurses when working with experienced nurses were determined to be feeling psychological pressure and having communication problems [10]. İleri et al. reported that the presence of a supportive relationship was found when novice nurses found, especially the mentor nurse approaches, to be caring and developmental and had positive experiences during the transition process; on the other hand, the presence of negative relationships was found when they were exposed to unkind behaviors of managers and senior colleagues and did not receive help [11].

Despite the well‐documented challenges faced by novice nurses, interpersonal competence remains a critical yet insufficiently assessed component of early professional development. The ability to accurately measure these competencies is essential for identifying educational needs, supporting transition programs, and improving retention and quality of care. However, there is a lack of valid and reliable measurement tools specifically adapted to assess professional interpersonal competence among nurses in the Turkish context. This gap limits both research and evidence‐based interventions aimed at strengthening early nursing practice. Therefore, this study aims to adapt and evaluate the psychometric properties of the Professional Interpersonal Competence Evaluation Scale developed by Sato et al. [12] for use in Turkish, thereby contributing a valid and reliable instrument to the national literature.

Research Questions:

1. Is the ‘Professional Interpersonal Competence Evaluation Scale’ for novice nurses a valid measurement tool for Turkish nurses?
2. Is the ‘Professional Interpersonal Competence Evaluation Scale’ for novice nurses a reliable measurement tool for Turkish nurses?

## 2. Methods

### 2.1. Design

This study is a methodological study conducted to adapt and evaluate the psychometric properties of the novice nurses’ Professional Interpersonal Competence Evaluation Scale in Turkish. The study was carried out in two stages: (1) translation and cultural adaptation of the scale and (2) evaluation of its validity and reliability.

### 2.2. Participants

Data were collected through online forms from nurses working in two hospitals in eastern Turkiye between October and December 2024. The study sample consisted of 450 novice nurses who met the inclusion criteria. In this study, “novice nurses” were defined as nurses with less than 3 years of professional clinical experience, in line with the literature. Accordingly, only nurses who had been working in clinical settings for less than 3 years were included in the study. Nurses with three or more years of experience or those not actively involved in clinical practice were excluded. The scale included 27 items.

In validity and reliability studies, it is recommended that the sample size be at least 5–10 times the number of items and be increased by 20% to account for potential data loss and sampling error [13]. Accordingly, the minimum required sample size was calculated as 135–270 participants (27 × 5–10).

In addition, previous methodological studies suggest that sample sizes of 300 or more are considered adequate for factor analysis [14–16]. Therefore, data collection forms were distributed to 625 nurses, of whom 500 responded (response rate: 80%). After excluding incomplete or invalid responses (*n* = 50), a total of 450 nurses were included in the final analysis.

### 2.3. Data Collection Tools

Data were collected with the sociodemographic form and the Professional Interpersonal Competence Evaluation Scale for new nurses. The sociodemographic form consisted of questions including information such as age, sex, marital status, educational background, years of working in hospitals and clinics, working style, and years of working in the unit.

The Professional Interpersonal Competence Evaluation Scale for novice nurses was developed by 2024 et al. with 27 statements in a 5‐point Likert style (1 = I Strongly Disagree, 5 = I Strongly Agree). The evaluation is based on the mean scores of items. Exploratory factor analysis (EFA) showed two factors: (1) core competencies as a new nurse (15 items) and (2) relationship‐building skills in health teams (12 items) explained 80% of the variance. Internal consistency reliability was 0.94 and 0.91 (factors), and overall scale reliability was 0.95. Item‐rest (I‐R) correlation values > 0.6 were considered acceptable.

## 3. Stage I: Cultural Adaptation and Revision

Initially, bilingual researchers fluent in both Turkish (their native language) and English‐speaking cultures translated the English version. Then, a third independent researcher compared the two translations with the original version. Three translators and researchers resolved inconsistencies. Secondly, a native bilingual translator translated the instrument back into English independently. Two linguists who had a PhD in nursing and over 10 years of experience compared the back‐translated scale independently. They did not suggest adding or removing any items and believed that the translated questionnaire was fully understandable for Turkish nurses.

In this process, translators ensured both linguistic equivalence and conceptual equivalence between the original and Turkish versions of the scale. Expert reviewers evaluated the semantic accuracy, clarity, and cultural appropriateness of the translated items. Discrepancies identified during translation and back‐translation were resolved through discussion among the research team to reach consensus.

Following translation, a pilot implementation was conducted with 40 nurses to assess clarity, comprehensibility, and cultural relevance of the items. Participants provided feedback on item wording and understandability. No item exceeded the predefined threshold for ambiguity, and therefore no modifications or deletions were required at this stage. This process ensured that the final Turkish version was both linguistically accurate and contextually appropriate for the target population.

Eighteen experts, including nursing professors experienced in nursing education, nursing administrators, and nursing educators, were invited to evaluate I‐CVI and S‐CVI to examine whether the scale and its items reflect novice nurses’ evaluations of professional interpersonal competence. After 4 weeks, 12 experts responded (participation rate: 66.6%). The relevance of the items was assessed on a 4‐point scale: 1 point (not valid at all), 2 points (invalid), 3 points (valid), and 4 points (very valid). They were also asked to suggest alternative wording for ambiguous items. The I‐CVI is the rate of the number of experts rating each item as 3/4 points out of the total number of experts. I‐CVI and S‐CVI were analyzed, and if the I‐CVI for an item was < 0.80 or the S‐CVI/Average was < 0.90, it was considered for modification/deletion [17]. In the second round involving five experts, four items were revised to align with the experts′ perspectives, although the S‐CVI/Average and I‐CVI met the validity criteria at 0.941 and 0.856.

### 3.1. Pilot Implementation

After content validity analysis, the 27‐item novice nurses’ Professional Interpersonal Competence Evaluation Scale for nurses was formed. The form was applied to 40 nurses as a pilot implementation in two state hospitals [18]. During the pilot implementation, if more than 20% of the participants rated an item in the scale as unclear, this item was re‐evaluated [19]. After the pilot implementation, no items were removed, and the final 27‐item Professional Interpersonal Competence Evaluation Scale for novice nurses was obtained.

## 4. Stage II: Evaluation of Scale Validity‐Reliability

### 4.1. Item Analysis

Items were evaluated using corrected item‐total correlations and factor loadings. Items with item–total correlation values below 0.40 or nonsignificant values (*p* > 0.05) were considered for removal [19]. After the removal of each item, Cronbach’s alpha value was recalculated. Ideally, the removal of an item should not lead to a substantial increase in the overall Cronbach’s alpha coefficient.

Content‐related evidence for validity was obtained from expert ratings. The content validity was considered acceptable when I‐CVI ≥ 0.78 and S‐CVI/Ave ≥ 0.80.

In line with contemporary psychometric recommendations (COSMIN and Messick’s unified validity framework), validity was conceptualized as a holistic construct supported by multiple sources of evidence rather than independent “types” of validity.

For EFA, the adequacy of the sample size for factor analysis was assessed using the Kaiser–Meyer–Olkin (KMO) test (KMO > 0.80), and the suitability of the sample data for factor analysis was assessed using Bartlett’s test of sphericity (*p* < 0.05) [20]. The factor structure and the nature of factors were analyzed using a common factor model (rather than principal component analysis) and oblique rotation (Direct Oblimin) to allow correlated factors, which provides more realistic parameter estimates.

All factor analyses were conducted on Pearson product–moment correlation matrices using maximum likelihood (ML) estimation in SPSS and AMOS. Although Likert‐type response formats represent ordinal data and polychoric correlations are often recommended, Pearson correlations and ML estimation were used in this study due to the 5‐point scale and the relatively large sample size (*n* = 450). Under these conditions, ML estimation has been shown to produce stable and acceptable parameter estimates in previous methodological studies.

However, this approach represents a methodological limitation, and future studies are recommended to re‐examine the factor structure using polychoric correlation matrices and robust ordinal estimation methods. Common factors were determined with eigenvalues > 1.00, and factor loads of 0.40 were employed to determine the factor nature of each item. If an item had a factor load < 0.4 or a cross‐load was present, the item was removed. The cumulative explained variance of all common factors must exceed 40.0% [21]. This approach integrates evidence for internal structure validity while aligning with the holistic validity framework and contemporary standards for health measurement instruments.

### 4.2. Statistical Analysis

Data were analyzed using SPSS version 27.0 and AMOS version 24.0. Participant characteristics were summarized using descriptive statistics, including frequencies and percentages. To examine the internal structure of the scale, EFA was conducted. In accordance with contemporary psychometric recommendations, a common factor model rather than principal components analysis was employed, and factors were extracted using the Principal Axis Factoring (PAF) method.

Multiple criteria were used to determine the number of factors, including eigenvalues greater than 1.0 (Kaiser criterion), inspection of the scree plot, and theoretical interpretability. Given the expected correlations among the dimensions of professional interpersonal competence, an oblique rotation (Direct Oblimin) was applied. This approach allows factors to correlate and provides more realistic parameter estimates. Sampling adequacy for factor analysis was assessed using the KMO measure and Bartlett’s test of sphericity.

To evaluate structural validity, the two‐factor solution obtained from the EFA was subsequently tested using confirmatory factor analysis (CFA). CFA was conducted using AMOS version 24.0 with the ML estimation method. Although the scale items were measured on a 5‐point Likert‐type ordinal scale, previous research indicates that ML estimation produces stable and unbiased results when the number of response categories is five or more, and the sample size is sufficient [22].

Model fit was evaluated using the chi‐square to degrees of freedom ratio (*χ* ^2^/df), the Comparative Fit Index (CFI), Tucker–Lewis Index (TLI), the Root Mean Square Error of Approximation (RMSEA), and the Standardized Root Mean Square Residual (SRMR). The total sample (*N* = 450) was not divided to preserve statistical power; the potential risk of overfitting associated with this decision is acknowledged as a limitation and discussed accordingly.

Evidence for convergent and discriminant validity was examined. Internal consistency reliability was assessed using Cronbach’s alpha, split‐half reliability, and corrected item–total correlations.

### 4.3. Ethical Considerations

The study was conducted in full accordance with the principles of the Declaration of Helsinki. Before data collection, all participants were informed in detail about the purpose, procedures, potential risks, and voluntary nature of the study. Both verbal and written informed consent were obtained from all participants. Participants were explicitly informed of their right to withdraw from the study at any time without any consequences.

Ethical approval for the study was obtained from the Scientific Research Publication Ethics Committee of Muş Alparslan University (approval number: 54; date: 06.02.2024). In addition, institutional permissions were secured from the administrations of the two state hospitals in which the pilot implementation and data collection phases were carried out. Throughout the study, participant confidentiality and anonymity were strictly maintained; no identifying personal information was collected, and all data were stored securely and used solely for scientific purposes.

## 5. Results

It was reported that 97.8% of the nurses included were aged < 30 years, 65.6% were female, 62% were single, and 82.4% had undergraduate education. It was reported that 60.7% of the nurses had been working for 0–1 year, 49.8% worked as day shifts, and 38.4% worked in emergency services (Table 1).

**TABLE 1:** Distribution of descriptive characteristics of nurses.

Features	*n* ^∗^	%
Age		
< 30	440	97.8
> 30	10	2.4
Gender		
Woman	295	65.6
Male	155	34.4
Marital Status		
Single	279	62.0
Married	171	38.0
Education		
High School	26	5.8
License	371	82.4
Above undergraduate	53	11.8
Experience of Working as a Nurse		
0–1 year	273	60.7
1–3 years	177	39.3
Mode of Operation		
Gündüz	172	38.2
Day‐shift	224	49.8
Shift	54	12.0
Unit worked in		
Internal	53	11.8
Surgery	36	8.0
Intensive care	119	26.4
Emergency service	173	38.4
Other	69	15.3

^∗^Number and percentage distributions.

### 5.1. Validity Findings

EFA and CFA were employed to examine the factors of the Professional Interpersonal Competence Evaluation Scale. KMO analysis was performed to determine whether the number of data was large enough for EFA, and Bartlett’s test was performed to determine whether the relationships between the variables were significant and whether they differed from zero. The KMO value was 0.929, and Bartlett’s sphericity test was significant (*χ* ^2^ = 6852.706, *p* < 0.001), indicating that the data were suitable for EFA. In line with these results, it can be argued that the data are suitable for factor analysis [23]. Factor loads were evaluated as proposed by Comrey and Lee [24] (values above 0.71: excellent, values between 0.63 and 0.71: very good, values between 0.55 and 0.63: good, values between 0.45 and 0.55: fair, and those between 0.32 and 0.45: poor). Tabachnick and Fidell [25] suggest that the minimum threshold employed to determine significant factor loads must be 0.32. Guidelines might help in deciding which variables to include for a particular factor, but the cut‐off employed to determine which loads must be included is at the discretion of the researcher.

### 5.2. Determining the Number of Factors

Multiple criteria were used to determine the number of factors. In this study, the Kaiser criterion was applied, and factors with eigenvalues greater than 1.0 were retained. In addition, the theoretical interpretability of the factor structure and inspection of the scree plot were considered as complementary decision criteria.

The eigenvalues were obtained from the correlation matrix, in which the diagonal elements were replaced by the squared multiple correlations of each variable [26, 27], a procedure used to estimate the communality of each variable [27, 28]. The Kaiser eigenvalue > 1 rule is a simple and widely used approach in determining the number of factors [26, 29, 30].

**FIGURE 1:**
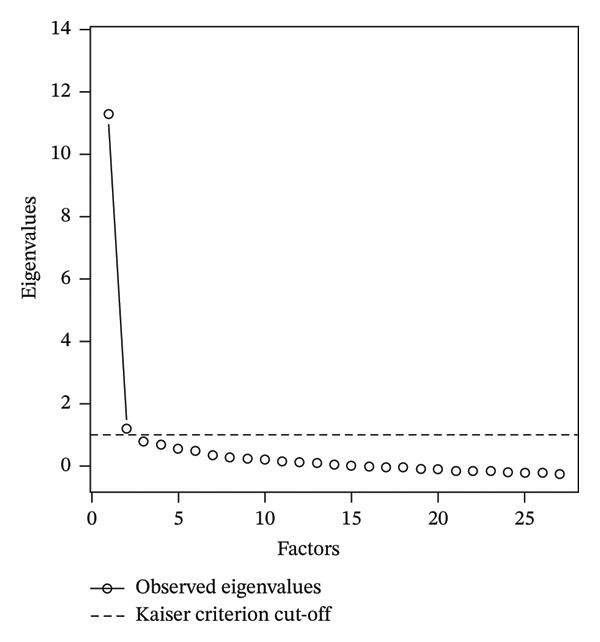
Scree plot with the Kaiser criterion.

Figure 1 presents both the scree plot and the Kaiser criterion used to evaluate the number of significant factors. The scree plot indicated a clear point of inflection, and together with the Kaiser criterion and theoretical interpretability, supported a two‐factor solution. Accordingly, two factors with eigenvalues greater than 1.0 were retained for EFA, and the resulting structure was found to be consistent with the conceptual framework of the scale.

### 5.3. Evaluation of Sample Size

The required sample size for EFA cannot be determined by a single rule; rather, it depends on the interaction of multiple factors, including item communalities, the number of items per factor, sample homogeneity, and the type of correlation matrix used [31–34]. The literature indicates that when communalities are at a moderate level (0.40–0.70), and each factor includes three to four items, a sample size of approximately *N* ≈ 200 is generally considered adequate. In contrast, models characterized by low communalities (approximately 0.30) and only three items per factor may require substantially larger samples, typically in the range of *N* = 400–500, to ensure stable factor solutions [35, 36].

In the present study, the majority of item communalities were at a moderate level (0.36–0.64), with only one item exhibiting a relatively low proportion of explained variance (*h* ^2^ ≈ 0.28; loading ≈ 0.36). The total sample size consisted of *N* = 450 participants, which falls within the upper range recommended in the literature and is sufficient to ensure a stable and replicable factor structure, even in the presence of items with lower communalities. Accordingly, the sample size provides strong support for the reliability of the factor solution and enhances the generalizability of the findings.

During the preliminary EFA, one item (Item 24) was removed from the scale due to cross‐loading behavior. Following this modification, the repeated factor analysis yielded a total explained variance of 44.485%. The EFA identified two factors with eigenvalues greater than 1. Factor 1 had an eigenvalue of 9.421 and accounted for 36.235% of the total variance, whereas Factor 2 had an eigenvalue of 2.145 and explained 8.250% of the variance. A summary of the factor analysis results is presented in Table 2.

**TABLE 2:** Eigenvalues, variance percentages, and cumulative percentages of factors.

Factor	Eigenvalue	% of variance	Cumulative (%)	Extraction sums of squared loadings (total)
1	9.421	36.235	36.235	8.912
2	2.145	8.250	44.485	1.654

The scale was accepted as two subdimensions in line with the original, and the factor pattern was found to be acceptable. The factor loads of all items related to this scale were found to be between 0.510 and 0.812 (Table 3).

**TABLE 3:** Professional Interpersonal Competence Assessment Scale.

Variable	Factor loading		Communality
	1	2	
A28	0.812		0.637
A30	0.745		0.613
A31	0.732		0.625
A32	0.718		0.514
A42	0.705		0.533
A41	0.688		0.560
A36	0.662		0.604
A27	0.655		0.605
A29	0.648		0.528
A34	0.632		0.561
A22	0.585		0.543
A35	0.510		0.273
A6		0.722	0.494
A9		0.705	0.562
A18		0.658	0.646
A5		0.642	0.454
A17		0.625	0.552
A3		0.618	0.498
A2		0.605	0.510
A14		0.598	0.440
A13		0.588	0.333
A15		0.565	0.422
A8		0.558	0.380
A4		0.552	0.395
A23		0.525	0.541
A11		0.502	0278

*Note:* Exploratory factor analysis.

The suitability of the 2‐factor structure of the Professional Interpersonal Competence Evaluation Scale, which emerged as a result of EFA, was tested with CFA (Table 4).

**TABLE 4:** Confirmatory factor analysis findings of the Professional Interpersonal Competence Assessment Scale.

Factor	Items	Estimate	S.E.	CR		AVE	
1	A6	0.549		0.90		0.41	
	A9	0.725	0.166				
	A18	0.825	0.166				
	A5	0.621	0.154				
	A17	0.766	0.157				
	A3	0.656	0.148				
	A2	0.676	0.167				
	A14	0.608	0.176				
	A13	0.521	0.153				
	A15	0.739	0.173				
	A8	0.622	0.162				
	A4	0.489	0.177				
	A23	0.425	0.164				
	A11	0.591	0.165				
2	A35	0.460		0.92		0.49	
	A22	0.715	0.223				
	A34	0.726	0.185				
	A29	0.692	0.247				
	A27	0.770	0.228				
	A36	0.673	0.212				
	A41	0.691	0.198				
	A42	0.731	0.210				
	A32	0.675	0.198				
	A31	0.792	0.169				
	A30	0.775	0.189				
	A28	0.732	0.214				

**Model**	**Chi-square (** **χ** ^2^ **)**	**df**	**χ** ^2^ **/df**	**RMSEA**	**CFI**	**TLI**	**SRMR**

One‐Factor Model	2145.12	299	7.17	0.118	0.824	0.802	0.092
Two‐Factor Model	1376.89	298	4.62	0.078	0.941	0.924	0.065
Acceptable Limit	—	—	≤ 5	≤ 0.08	≥ 0.90	≥ 0.90	≤ 0.08

*Note:* Standardized regression coefficients, AVE, CR values of the Professional Interpersonal Competence Assessment Scale.

CFA was conducted to validate the two‐factor structure consisting of 26 items as identified by the EFA. All analyses were performed using the ML estimation method. Considering the five‐point Likert‐type response format and the sample size, ML estimation was deemed appropriate and capable of providing stable and unbiased parameter estimates [22].

To evaluate the model fit, multiple goodness‐of‐fit indices were examined. The results indicated that the two‐factor model demonstrated an acceptable fit to the observed data. Furthermore, to assess the discriminant validity of the factor structure and to evaluate the appropriateness of computing a total scale score, a one‐factor model was compared with the two‐factor model.

According to the analysis results, the fit indices of the two‐factor model (*χ* ^2^/df = 4.62; RMSEA = 0.078; CFI = 0.94; TLI = 0.92) indicated a significantly better fit compared to the one‐factor model and fell within acceptable thresholds [37, 38]. Examination of the factor loadings revealed that all items loaded significantly on their respective factors (*p* < 0.001), with standardized loadings ranging from 0.36 to 0.82, indicating adequate to strong associations with the latent constructs.

**FIGURE 2:**
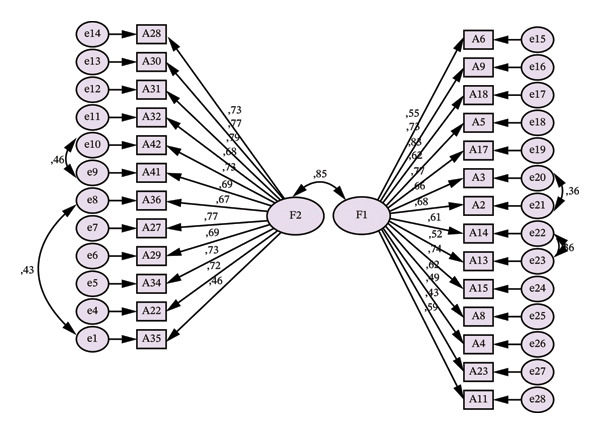
Path diagram of the Professional Interpersonal Competence Assessment Scale.

To evaluate the essential unidimensionality of the scale, the explained common variance (ECV) was calculated and found to be 0.64. The literature suggests that ECV values exceeding 0.60 support the presence of a dominant general factor despite the multidimensional structure of the scale, thereby justifying the interpretation of a total score [39]. This finding confirms that the scale can be meaningfully used both in terms of its subscale scores and its overall total score. The path diagram of the model is given in Figure 2. Average variance extracted (AVE) and composite reliability (CR) values were analyzed to confirm the convergent and discriminant validity of the Professional Interpersonal Competence Evaluation Scale. AVE values analyzed for each factor ranged between 0.41 and 0.49. CR values were found to be between 0.91 and 0.92 [40, 41].

### 5.4. Reliability Analyses

It is stated that the minimum value needed for the item‐total score correlation to be adequate must be 0.20, and items with this value between 0.20 and 0.30 can be included in the measurement tool if needed [42]. When the Professional Interpersonal Competence Evaluation Scale was analyzed, it was reported that the item‐total score correlation was between 0.353 and 0.753. To evaluate the internal consistency of the scale, Cronbach’s alpha (*α*) coefficient was calculated alongside McDonald’s omega (*ω*), which provides more accurate reliability estimates when the assumption of tau‐equivalence is violated [43]. For the total scale, Cronbach’s alpha was 0.945, and McDonald’s omega was 0.948. The omega coefficients for the two subscales were 0.922 and 0.904, respectively. The fact that all reliability coefficients exceeded the recommended threshold of 0.70, together with the close correspondence between alpha and omega values, indicates a high level of internal consistency and suggests that the items reliably and consistently represent the underlying construct.

### 5.5. Split‐Half Reliability

Based on the split‐half reliability analysis, the Spearman–Brown corrected correlation coefficient was 0.769, and the Guttman split‐half reliability coefficient was 0.869. In addition, the Cronbach’s alpha coefficients for each half of the scale were found to be satisfactory (Table 5). Collectively, these findings indicate that the 26‐item Professional Interpersonal Competence Assessment Scale demonstrates a high level of internal structural consistency.

**TABLE 5:** Findings related to split‐half reliability of the Professional Interpersonal Competence Assessment Scale.

Reliability statistics			
Cronbach’s Alpha	Part 1	Value	0.900
		N of Items	13^a^
	Part 2	Value	0.907
		N of Items	13^b^
	Total N of Items		26
Correlation Between Forms			0.769
Spearman–Brown Coefficient	Equal Length		0.869
	Unequal Length		0.869
Guttman Split‐Half Coefficient			0.869

^a^The items are: A2, A3, A4, A5, A6, A8, A9, A11, A13, A14, A15, A17, A18.

^b^The items are: A22, A23, A25, A27, A28, A29, A30, A31, A32, A34, A36, A41, A42.

## 6. Discussion

The Turkish validity‐reliability scale that was developed by Sato et al. (2024) to assess professional interpersonal competence for novice nurses, consisting of 27 items and 2 subscales, was evaluated. Validity is whether an instrument measures what it aims to measure [44]. The consensus of experts on the clarity and applicability of items in psychometric instruments is accepted as a criterion for content validity [45]. In the literature, it is recommended that the translation must be carried out by two or more independent people who are competent in the scale’s original language and the adapted language and who understand cultural and linguistic features [46]. In the present study, the original version was translated by independent translators who were fluent in English and Turkish. After the Turkish translation, the content validity and its items were analyzed. As stated in the literature [47].

It is expected that the I‐CVI value is ≥ 0.80, but according to Lynn (1986), this value must not be less than 0.78. In the present study, the I‐CVI value was 0.856, which is above the values needed in the literature, indicating that the scale is suitable for the Turkish population. The construct validity was analyzed with EFA, which is a statistical technique employed to explain a measurement with a small number of factors to measure the same construct or feature [48]. In the present study, KMO and Bartlett tests were also used. A KMO value greater than 0.5 is considered adequate to accept that the data has a normal distribution and is, for this reason, considered suitable for EFA. A significant Bartlett’s test of sphericity also shows that the data are adequate for factor analysis [49]. In the present study, the KMO value for the scale was 0.929, and the Bartlett’s test of sphericity was *χ* ^2^ = 6852.706, *p* < 0.001. Based on these values, it is seen that the data are suitable for factor analysis.

Following the EFA, CFA was used. The goodness‐of‐fit indices of the model were evaluated based on the criteria accepted in CFA and found to be acceptable [50]. There are various modalities, such as factor analysis, internal consistency analysis, hypothesis testing, convergent validity, structural equation modeling, multivariate matrix, and pattern matching theory, to evaluate construct validity [51]. The construct validity of the Professional Interpersonal Competence Evaluation Scale was examined using EFA and CFA. The EFA revealed a two‐factor structure, which was confirmed by CFA. Goodness‐of‐fit indices indicated an acceptable model fit. Convergent and discriminant validity were assessed using appropriate statistical methods. The findings provide evidence supporting the construct validity of the 26‐item, two‐factor scale. Factor 1 explained 36.235% of the variance (eigenvalue = 9.421), while Factor 2 explained 8.250% of the variance (eigenvalue = 2.145).

The two‐factor solution explained approximately 44% of the total variance. Although this level of explained variance is considered acceptable in social and behavioral sciences, it is lower than that reported in the original scale development study. This difference may be attributed to cultural differences between the original context and the Turkish sample, as well as variations in sample characteristics, clinical experience, and data collection settings. In addition, cross‐cultural adaptation processes may introduce subtle differences in item interpretation, which can influence factor loadings and explained variance. Despite these differences, the obtained variance remains within acceptable limits and, together with the factor structure and fit indices, supports the construct validity of the scale in the Turkish context.

The number of factors was determined using multiple criteria, including the Kaiser criterion (eigenvalues > 1), scree plot inspection, and theoretical interpretability. These criteria were evaluated together to ensure a robust and theoretically consistent factor solution. No additional factor retention methods, such as parallel analysis or minimum average partial (MAP) tests, were conducted in this study. This approach is commonly used in scale adaptation studies; however, the absence of additional empirical retention methods may be considered a limitation when interpreting the stability of the factor structure.

Reliability refers to how consistent a variable is with what it measures [52]. Cronbach’s *α* value is widely employed by researchers in scale adaptations and shows the items represent a homogenous structure [51]. If Cronbach’s *α* value is between 0.80 and 1.00, the scale can be considered highly reliable [53]. Cronbach’s *α* value was 0.945. Based on these results, it can be argued that the 26‐item 2‐factor scale is reliable. During the adaptation into Turkish, one item was removed, and no changes were made in the factor dimensions. This can be attributed to some similarities between the Japanese society and the Turkish society in which the scale was first developed. These similarities enabled the scale to be successfully adapted without any significant changes in its structure. This result supports the validity and applicability of the scale. This can be attributed to the similarities between the Japanese society and the Turkish society in which the scale was first developed. These similarities enabled a smooth adaptation without the need for serious changes in the structure of the scale. This result emphasizes the importance of the scale.

While the present study provides a culturally adapted and psychometrically sound instrument, it may also offer practical implications for nursing management and workforce development. In particular, the scale could assist nurse managers in assessing interpersonal competencies, identifying potential areas for improvement, and informing interventions aimed at supporting team communication, retention, and quality of care.

### 6.1. Limitations

This study has several limitations that should be considered when interpreting the findings. First, the sample consisted of nurses working in only two public hospitals who voluntarily agreed to participate, which may limit the generalizability of the results to other settings and populations. In addition, the predominantly female composition of the sample (65.6%) may further restrict the representativeness of the findings.

Second, a methodological limitation relates to the estimation approach used in factor analyses. The analyses were conducted based on Pearson correlations and ML estimation rather than polychoric correlations with estimation methods specifically designed for ordinal data. Although the use of a 5‐point Likert‐type scale and a relatively large sample size (*N* = 450) may have mitigated potential bias, future studies are recommended to re‐examine the factor structure using polychoric correlation matrices and robust ordinal estimation techniques.

Third, the number of factors was determined using the Kaiser criterion and scree plot inspection. However, more advanced and statistically robust factor retention methods, such as parallel analysis or the MAP test, were not employed. This may limit the strength and stability of the factor retention decision.

Finally, exploratory and CFA were conducted on the same sample, which may increase the risk of model overfitting. Future research should validate the factor structure using independent samples to enhance the robustness and generalizability of the findings.

## 7. Conclusion

The results show that the Professional Interpersonal Competence Evaluation Scale for Novice nurses, which was tested for reliability and validity in the Japanese population, is a reliable and valid evaluation tool for Turkiye. These findings indicate that the Professional Interpersonal Competence Evaluation Scale for novice nurses can be used in Turkish society and suggest that it can be used effectively to measure this society.

In addition, the availability of a valid and reliable Turkish version of the scale provides a practical tool for nursing managers to assess interpersonal competencies among novice nurses, identify areas requiring development, and support workforce performance and team communication in clinical settings. From an educational perspective, the scale may be used by nursing educators to evaluate students’ or newly graduated nurses’ interpersonal competence, guide curriculum development, and inform targeted training programs aimed at strengthening communication, collaboration, and professional integration into clinical practice.

## Author Contributions

Cemal Özalp: conceptualization; methodology; data curation; formal analysis; investigation; writing–original draft; writing–review and editing; visualization; and supervision.

## Funding

No funding was received for this study.

## Disclosure

The author confirms that the manuscript has been read and approved and that there are no other individuals who meet the authorship criteria.

## Ethics Statement

Ethical approval was obtained from the Scientific Research Publication Ethics Committee of Muş Alparslan University (Approval No: 06.02.2024‐54).

## Conflicts of Interest

The author declares no conflicts of interest.

## Data Availability

The data that support the findings of this study are available on request from the corresponding author. The data are not publicly available due to privacy or ethical restrictions.
